# Heteronuclear Complexes of Hg(II) and Zn(II) with Sodium Monensinate as a Ligand

**DOI:** 10.3390/molecules29133106

**Published:** 2024-06-29

**Authors:** Ivayla Pantcheva, Nikolay Petkov, Elzhana Encheva, Stiliyan Kolev, Svetlana Simova, Aleksandar Tsanev, Petar Dorkov, Angel Ugrinov

**Affiliations:** 1 Faculty of Chemistry and Pharmacy, Sofia University “St. Kliment Ohridski”, 1164 Sofia, Bulgaria; eencheva@ipc.bas.bg (E.E.); stiliiankolev@gmail.com (S.K.); 2 Institute of Physical Chemistry, Bulgarian Academy of Sciences, 1113 Sofia, Bulgaria; 3 Institute of Organic Chemistry with Centre of Phytochemistry, Bulgarian Academy of Sciences, 1113 Sofia, Bulgaria; svetlana.simova@orgchm.bas.bg; 4 Institute of General and Inorganic Chemistry, Bulgarian Academy of Sciences, 1113 Sofia, Bulgaria; tsanew@abv.bg; 5 Research and Development Department, Biovet Ltd., 4550 Peshtera, Bulgaria; p_dorkov@biovet.com; 6 Department of Chemistry and Biochemistry, North Dakota State University, Fargo, ND 58108-6050, USA; angel.ugrinov@ndsu.edu

**Keywords:** polyether ionophore, mixed-metal mixed-ligand complexes, spectral features

## Abstract

The commercial veterinary antibiotic sodium monensinate (MonNa) binds mercury(II) or zinc(II) cations as thiocyanate [Hg(MonNa)_2_(SCN)_2_] (**1**) or isothiocyanate [Zn(MonNa)_2_(NCS)_2_] (**2**) neutral coordination compounds. The structure and physicochemical properties of **1** and **2** were evaluated by the methods of single crystal and/or powder X-ray diffraction, infrared, nuclear magnetic resonance, X-ray photoelectron spectroscopies, and electrospray-mass spectrometry. The primary cores of the two complexes comprise HgS_2_O_2_ (**1**) and ZnN_2_O_2_ (**2**) coordination motifs, respectively, due to the ambidentate binding modes of the SCN–ligands. The directly bound oxygen atoms originate from the carboxylate function of the parent antibiotic. Sodium cations remain in the hydrophilic cavity of monensin and cannot be replaced by the competing divalent metal ions. Zinc(II) binding does not influence the monensin efficacy in the case of *Bacillus cereus* and *Staphylococcus aureus* whereas the antimicrobial assay reveals the potential of complex **2** as a therapeutic candidate for the treatment of infections caused by *Bacillus subtilis*, *Kocuria rhizophila*, and *Staphylococcus saprophyticus*.

## 1. Introduction

**Figure 1:**
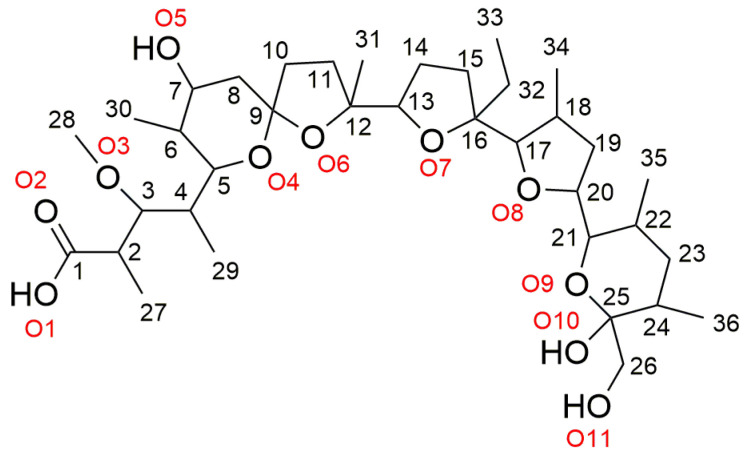
Chemical structure and numbering scheme of monensic acid.

The early history of polyether antibiotics dates back to 1951, when nigericin and X-537A (lasalocid A) were isolated from *Streptomyces spp*. [1,2]. These were found to exhibit activity against Gram-positive microorganisms and mycobacteria but were ineffective against Gram-negative bacteria and were not classified as polyether compounds at the time. Years later, in 1967, the structure of monensic acid (MonH, Figure 1) has been proved to be the first representative of polyether antibiotics, and its isolation, fermentation, chemical properties, anticoccidial activity, and mode of action have become known [3,4,5,6,7,8,9]. Today, more than 120 natural polyether ionophores have been reported [10], and the main use of some of them is for the control of coccidiosis in agriculture. Statistics show that the most used antibiotics in veterinary medicine are lasalocid, monensin, salinomycin, narasin, and maduramycin [11].

The striking feature of the ionophores is the selective binding to certain metal ions, which is largely determined by the length of their polyether chain. These antibiotics adopt a cyclic conformation where the O-donor atoms are internally oriented and form a hydrophilic cavity capable of accommodating a water molecule or a positively charged cation. Some of the ionophores form neutral coordination compounds with monovalent metals and are therefore known as monovalent polyether antibiotics [12,13,14,15,16,17,18,19,20]. On the other hand, the biological action of monensin, in particular, is sensitive to environmental conditions: it depends on pH in *Streptomyces bovis* [21] and is influenced by the presence of closely located Mg(II) cations [22]. The latter can be at least partially explained by the potential interaction of this monovalent polyether antibiotic with divalent metal ions to form new metal(II)-containing complex species [23,24]. To prove this hypothesis, we began an extensive study of monensin’s ability to bind metal cations with different oxidation states [25,26,27,28,29,30].

In this paper, we discuss the coordination behaviour of the commercially available sodium monensinate (MonNa) in the presence of bio (Zn(II)) and toxic (Hg(II)) ions. As will be seen, the reaction of MonNa with metal(II) thiocyanates leads to the isolation of new heteronuclear monensinates, the structures of which have been solved by X-ray diffraction (XRD) methods. The binding mode of the ligands has also been evaluated by infrared (IR) and nuclear magnetic resonance (NMR) spectroscopies. The formation of new mixed-metal species of the compositions [Hg(MonNa)_2_(SCN)_2_] and [Zn(MonNa)_2_(NCS)_2_] confirms the potential of sodium monensin to participate in additional complexation reactions and the data obtained enrich its coordination chemistry.

## 2. Results

Reactions of sodium monensinate with metal(II) thiocyanates lead to the isolation of crystalline solids with the composition [Hg(MonNa)_2_(SCN)_2_] (**1**) and [Zn(MonNa)_2_(NCS)_2_] (**2**). The structure of **1** is solved by a single crystal X-ray diffraction method, and the coordination mode of the ligands in **1** and **2** is additionally assessed by IR and NMR spectroscopies. The structure of **2** is also confirmed with powder XRD. The mixed-metal complexes exhibit similar structures, with hosted sodium cations that cannot be removed from the antibiotic cavity. The overall neutral character of the reported coordination species is secured by two monodentate antibiotic monoanions and two thiocyanates enveloping the primary coordination core of the divalent metal ions. The SCN^−^–ligands possess different binding patterns in the two complexes—they are S-bonded to Hg(II) and N-bonded to Zn(II) cations. The complex species reported herein are the first examples of diamagnetic heteronuclear complexes of monensin.

### 2.1. Description of the Crystal Structure of Complex ***1***

**Figure 2:**
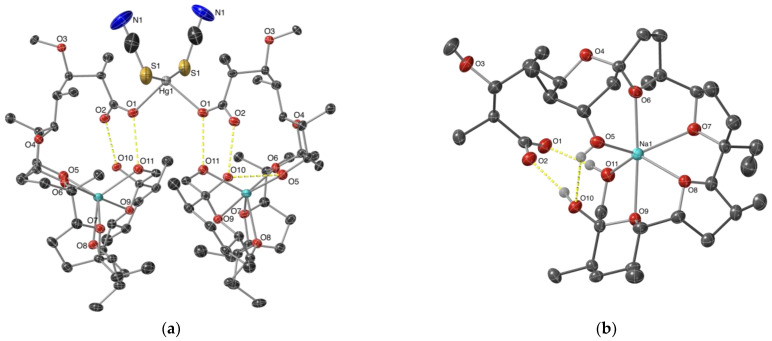
ORTEP diagram (50% probability level) of (**a**) complex **1** and (**b**) sodium monensinate core in **1**. Protons are omitted for clarity. Colour code: C—dark grey, O—red, N—dark blue, S—yellow, Na—light blue, Hg—grey, H bonds—yellow dash.

**Figure 3:**
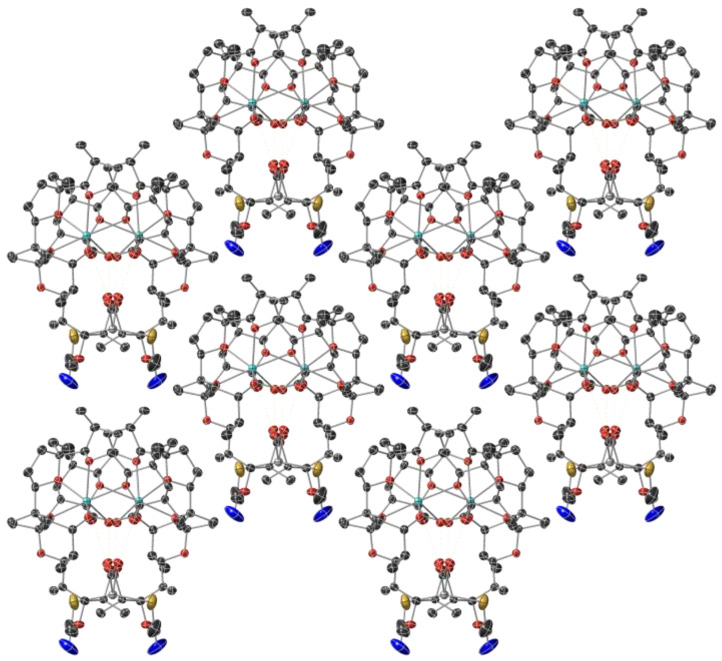
Crystal packing of complex **1** along the c-axis. Protons are omitted for clarity. Colour code: C—dark grey, O—red, N—dark blue, S—yellow, Na—light blue, Hg—grey.

The ORTEP diagram and crystal packing of complex **1** are plotted in Figure 2a and Figure 3, respectively. Each Hg(II) cation is four-coordinated by two sodium monensinates and two thiocyanates in a rather distorted tetrahedral geometry with bond angles around the metal centre varying from 91.4 to 159.1° (Table 1). The Hg-O bonds arise from the interaction between the metal cation and the monodentate carboxylate function of the antibiotic anions, with a distance typical of Hg(II)-carboxylates [31,32]. The length of the Hg–S bonds is similar to that in the HgS_2_O_2_ coordination motif [33,34], but is significantly shorter compared with Hg(II)-thiocyanate complexes bearing nitrogen- or sulphur-comprising co-ligands [35,36,37,38,39]. Representative cases of Hg-O and Hg-S distances in various mercury(II) coordination species are shown in Table 2. To the best of our knowledge, the structure of [Hg(MonNa)_2_(SCN)_2_] is the first example of a mercury(II) dicarboxylate–dithiocyanate complex. As commonly observed in Hg(II)–thiocyanate compounds, the SCN-groups are linear, the Hg-S-C angles are close to 100°, and the C-S and C-N bond lengths are 1.65 and 1.16 Å, respectively [40,41].

Sodium cations cannot be replaced by Hg(II) ions and remain accommodated into the hydrophilic space of monensinate ligands. The metal(I) centre is coordinated to four ether (O6, O7, O8, O9) and two hydroxyl (O5, O10) donor atoms in a highly distorted octahedral geometry (Table 1, Figure 2b). Na-O bonds range within 2.33–2.46 Å; the observed distances are in close agreement with previously reported data on sodium monensinate [42,43] and its heteronuclear complexes that contain Co(II) or Mn(II) cations and chloride co-ligands [44]. The carboxylate and hydroxyl ends of monensinate are interconnected with each other by O11-H ··· O1 (2.700 Å) and O10-H ··· O2 (2.631 Å) intramolecular hydrogen bonds; in addition, the antibiotic anions are stabilized by a third H-bond O5-H ··· O10 (2.921 Å). Neither additional and obvious intermolecular interactions such as H-bonds between the ligands, nor the inclusion of solvent molecules that may serve as bridge(s) for H-bonds are observed in the crystal packing of complex **1** (Figure 3).

**Table 1:** Selected bond lengths (Å) and angles (°) for [Hg(MonNa)_2_(SCN)_2_], **1**.

Hg-O1	2.429(4)	Na-O5	2.328(5)
Hg-S1	2.375(2)	Na-O6	2.350(5)
O1-Hg-O1 ^11^	102.60(20)	Na-O7	2.447(5)
S1^1^-Hg-S1	159.13(11)	Na-O8	2.400(8)
S1-Hg-O1	91.42(12)	Na-O	2.457(5)
S1^1^-Hg-O1	101.64(11)	Na-O11	2.339(5)

**Table 2:** Mercury-donor atom bond distances in Hg(II) complexes bearing variable coordination motifs.

Ligand/Complex	Core Unit	Bond Length [Å]			Refs.
		Hg-O	Hg-N	Hg-S	
quinoline-2-carboxylic acid/[HgL_2_X], X = H_2_O, EtOH	HgN_2_O_3_	2.31–2.52	2.19–2.26	−	[39]
picolinic acid/[HgL_2_Cl]	HgN_2_O_2_Cl	2.49–2.54	2.12–2.35	−	[40]
pyridine-2-thione/[HgL_2_]	HgS_4_	−	−	2.56–2.68	[43]
biquinoline/[HgL_2_(SCN)_2_]	HgN_2_S_2_	−	2.30–2.50	2.44–2.47	[44]
bipyridyl-based carbazoles/[HgL_2_(SCN)_2_]	HgN_2_S_2_	−	2.30–2.45	2.44–2.45	[45]
nicotinamide/[HgL_2_(SCN)_2_]	HgN_2_S_2_	−	2.33–2.39	2.42–2.46	[46]
4,5-diazafluoren-9-one/[HgL_2_(SCN)_2_]	HgN_2_S_2_	−	2.48	2.4	[47]
3-(2-chlorophenyl)-2-sulfanylpropenoic acid					[41]
[HgL_2_]^2−^	HgO_2_S_2_	2.55–2.61	−	2.34	
[HgL(HL)]^−^	HgO_2_S_2_	2.54–2.67	−	2.34	
[Hg(HL)_2_]	HgO_2_S_2_	2.65	−	2.36	
thiol (L′) & carboxylic acid (L″)/[HgL′_2_L″]	HgO_2_S_2_ *	2.46–27		2.33–2.41	[42]

* bidentate carboxylate function.

### 2.2. Spectral Characterization of ***1*** and ***2***

**Figure 4:**
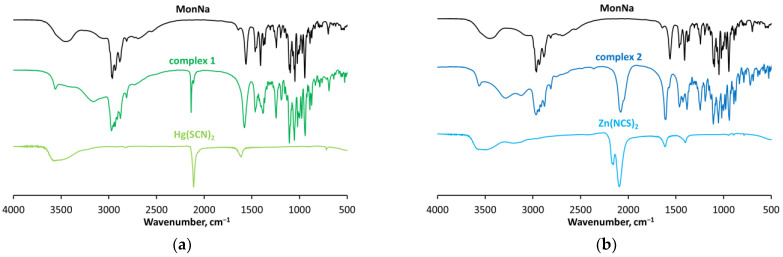
IR spectra of (**a**) MonNa, complex **1** and Hg(II) thiocyanate and of (**b**) MonNa, complex **2** and Zn(II) isothiocyanate.

The IR spectrum of MonNa (Figure 4a,b) consists of two bands at 1560 cm^−1^ and 1408 cm^−1^ (Δ = 152 cm^−1^) attributed respectively to the ν_COO_^asym^ and ν_COO_^sym^ of the carboxylate function that is not involved in direct interaction with sodium cations. The stretching vibrations of the hydroxyl groups of the antibiotic appear as a broad signal in the range of 3550−3300 cm^−1^, accompanied by a low-intensity band at 1640 cm^−1^ assigned to δ_OH_ [45,46,47,48]. The parent mercury(II) salt exists in a pure thiocyanate form, as evidenced by its strong absorption band assigned to the C≡N bond (2100 cm^−1^), complemented by a weak C-S stretch appearing at 718 cm^−1^ (Figure 4a). In Zn(SCN)_2_, most of the zinc(II) ions are involved in the formation of metal-nitrogen bonds, apparent from the intense broad band at 2092 cm^−1^ (ν_CN_) and the weak absorption at 893 cm^−1^ (ν_CS_), while the rest exist in a bridge-like M-SCN-M structure with characteristic stretches at 2156 cm^−1^ and 784 cm^−1^, related to ν_CN_ and ν_CS_, respectively [49,50,51] (Figure 4b).

The IR spectra of **1** and **2** (Figure 4a,b) show several characteristic bands assigned to the main donor groups participating in complex formation. The asymmetric and symmetric stretches of the carboxylate function are observed at 1574/1405 cm^−1^ (**1**, Δ = 169 cm^−1^) and at 1640/1430 cm^−1^ (**2**, Δ = 210 cm^−1^), respectively, assuming its monodentate coordination mode to the heavy metal(II) centre. The presence of thiocyanate ligands is evident from the absorption bands detected in the range of 2200−2000 cm^−1^. The C≡N frequency in **1** appears as a strong sharp signal at 2135 cm^−1^, indicative of the formation of a Hg-thiocyanate bond (Hg-SCN), while the spectrum of **2** consists of an intense but much broader band at 2070 cm^−1^ revealing the presence of Zn-isothiocyanate (Zn-NCS) interaction [52]. The bands at 3555 (**1**) and 3562 cm^−1^ (**2**) are assigned to O11-H groups participating only in the formation of a hydrogen bond with the carboxylate O2-oxygens, which are not involved in the formation of the first coordination sphere. The rest of the signals, namely those at 3158 cm^−1^ (Hg(II)complex) and 3293/3110 cm^−1^ (Zn(II)species), are typical of monensinate OH-functions involved in donor–acceptor interactions with sodium cations and/or H bond(s).

The ^1^H- and ^13^C-NMR spectra of the diamagnetic Hg(II) (**1**) and Zn(II) (**2**) mixed-metal monensinates were also studied using NMR spectroscopy. The chemical shifts of the ^1^H- and ^13^C-resonances in MonNa and **1**–**2** are presented in Section 2.2. and Table 3, respectively, following the numbering shown in Figure 1. The ^1^H- and ^13^C-NMR spectra of the studied compounds **1**–**2** are shown in Figures S1 and S2.

The NMR spectra of complex **1** in solution do not change significantly compared with those of sodium monensinate. Most of the carbon signals in the ^13^C-NMR spectrum of **1** retain their positions, with minor exceptions such as the upfield shifts for 2C and 3C atoms by 0.2 ppm. The observed spectra of **1** show that this complex is not sufficiently stable in chloroform solution and does not hold its molecular form in an identical manner to that in the solid state. On the contrary, the ^13^C-NMR spectrum of complex **2** is sufficiently different from that of MonNa. For carbon atom 1C, a very weak broad peak is observed, shifted downfield by 0.6 ppm compared with MonNa. Additionally, another low-intensity signal at 135.2 ppm indicates the presence of the isothiocyanate ligand. 2C and 3C atoms that are located close to the metal cation differ by 0.9 ppm upfield and the signals of all carbon atoms directly bonded to the oxygens of the polyether chain are broadened, most likely due to O-participation in numerous intramolecular bonds. The observed difference in several NMR resonances between complex **2** and MonNa indicates that the Zn(II) heteronuclear complex does not dissociate and retains its structure both in the solid state and in solution.

The ESI-MS+ spectra of complexes **1** and **2** (Figures S3 and S4, respectively) consist mainly of peaks assigned to [MonNa]H^+^ (693.42 *m*/*z*), [MonNa]Na^+^ (715.40 *m*/*z*) and [MonNa]_2_Na^+^ (1407.81 *m*/*z*), revealing the structure’s breakdown of the two neutral coordination species into their key compartments—MonNa (observable) and Hg(SCN)_2_/Zn(NCS)_2_ (not observable). The very low-intensity peak in the spectrum of **1** at 1725.74 *m*/*z* is assigned to the molecular ion of the parent complex [Hg(MonNa)_2_(SCN)_2_]Na^+^, thus confirming its existence and its following dissociation under ESI conditions.

### 2.3. Structure Elucidation of Complex ***2***

**Figure 5:**
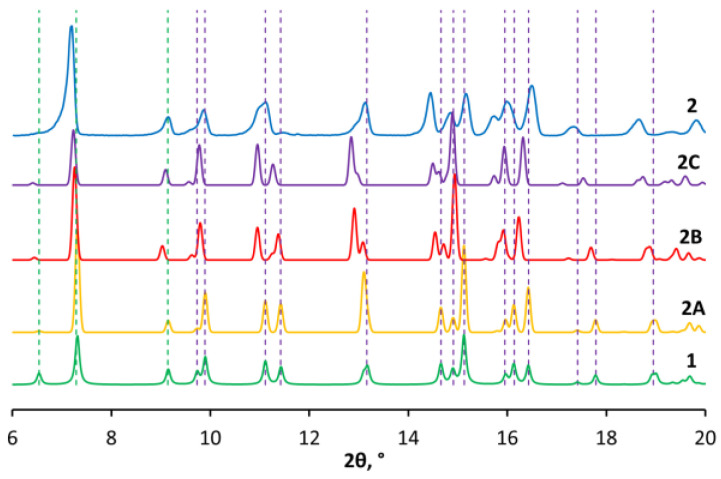
Powder XRD of complex **1**, modelled species **2A**–**C** and complex **2** within 6–20°.

Based on the spectral properties of Zn(II) complex **2** we suggest that its structure resembles that of [Hg(MonNa)_2_(SCN)_2_] (**1**), with one major difference, which is due to the coordination mode of the thiocyanate as ambidentate ligand. The main evidence for the formation of zinc–isothiocyanate (Zn-NCS) bonds in **2** is apparent from its IR spectral characteristics. Unfortunately, we failed to grow single crystals suitable for X-ray diffraction; however, we obtained polycrystalline material of reasonable quality in order to conduct powder XRD analysis (Figure 5, blue). The powder pattern of **2** was compared with that calculated for complex **1** (green), and the significant similarities found suggest identical space groups between complexes **1** and **2**.

Following the last observation and spectral properties of **2**, we assumed that **1** and **2** are isostructural, where Hg(SCN)_2_ is replaced by a Zn(NCS)_2_ fragment. It is well known from XRD theory that the position and numbers of observed peaks in powder diffraction experiments are based on identical cells parameters, while their intensity is a function of the cell contents. We tried to go further. We simulated the powder pattern of the assumed Zn structure (Zn connected to N in thiocyanate anion) and compared it with the experimentally observed powder patter of the Zn–compound. Thus, we replaced the mercury(II) cations into the crystal structure of **1** with zinc(II) ions, preserving the S-binding of thiocyanate ligands, and simulated the corresponding pattern before (**2A**, yellow) and after (**2B**, red) optimization. In the final step, the sulphur and nitrogen atoms of the SCN groups were exchanged to model the Zn-NCS interaction (**2C**, purple, after optimization). The description of the protocol for powder diffraction patterns and crystal structure simulations of Zn(II) complex is provided in the Supplementary Materials (Figure S5 and Tables S1 and S2).

In all five diffraction patterns, a complete conservation of the number of reflections can be seen, which is due to the similar unit cell of the complexes. The peaks of the studied species **1**, **2A**–**C** and **2** retain their positions up to 10°, maintaining the same intensity ratio. The observed splitting at 11.1° and 11.4° in **1** and **2A** gradually shifts from **2B** to **2C** towards lower 2θ values (11.0° and 11.3°) to better fit the broad and clearly composite reflection of **2** in this position. An interesting result is evident for the reflection at 13.2°—in **1** it appears as an asymmetric peak of relatively low intensity, while in **2A** it is observed as a symmetric and more intense reflection. Optimization of the S-bound construct (**2B**) raises an additional peak at 12.9°. Replacement of sulphur with nitrogen as a donor atom in the modelled Zn(II) structure **2C** further diminishes the original reflection at the expense of the new one. Although the intensity of the two peaks is reversed in **2C** compared with **2**, their position corroborates well the broad asymmetric reflection observed in the diffractogram of the isolated complex **2**. The remaining patterns in the range 14−17° deviate to some extent between all species within 0.1−0.3°; however, the overall peaks comparison reveals the close similarity between the structures of [Hg(MonNa)_2_(SCN)_2_] (**1**) and [Zn(MonNa)_2_(NCS)_2_] (**2**). In conclusion, the negligible differences, mostly in the peak intensities and shoulders in the XRD patterns of **1** and **2**, can be explained by the described chemical changes in the contents of the unit cell. In addition, the temperature dependence of the unit cell parameters must be taken into account, since the single-crystal X-ray diffraction was measured at 107 K and powder XRD at 298 K. Shrinkage of the unit cell will lead to a slight shift in the position of peaks.

To further examine the ambidentate properties of SCN-anions in Hg(II) and Zn(II) mixed-ligand complexes, we also studied the behaviour of **1**–**2** employing XPS. The full scan of both XPS spectra presented in Figure S6a shows typical peaks at 284.9 eV, 531.9 eV and 1070.7 eV, attributable to carbon (C1s), oxygen (O1s), and sodium (Na1s), respectively. In addition, the spectrum of **1** consists of signals characteristic of mercury(II) (Hg4f, 101.0 eV), sulphur (S2p, 163.0 eV) and nitrogen (N1s, 398.5 eV), and that of **2** comprises peaks at 162.4 eV, 398.4 eV and 1021.9 eV assigned respectively to sulphur (S2p), nitrogen (N1s) and zinc(II) (Zn2p) [53].

The high-resolution spectra (HRS) of C1s (Figure S6b) for Hg(II) and Zn(II) complexes were deconvoluted into three peaks at 284.7 eV, 286.1 eV and 288.5 eV, belonging to carbon electrons respectively involved in the formation of C-C, C-O, and COO^−^-bonds. The binding energy of O1s appears as a single symmetric peak attributable to C-OH and C-O-C. The equivalent shape, position and intensity of carbon and oxygen BE confirm the identity of the antibiotic ligand (both qualitatively and quantitatively) involved in the structure of coordination species **1** and **2**. HRS of Hg4f (Figure S6c) and Zn2p (Figure S6d) encompass peaks at 101.0 eV and 1021.6 eV, respectively, which confirm the expected presence of Hg(II) and Zn(II) cations in the studied mixed-metal monensinates [54,55,56,57]. 

The binding energy of S2p electrons (Figure S6e) is observed as a single peak with FWHM = 3.0 eV (**1**) and FWHM = 2.5 eV (**2**), respectively, while the signal of N1s in **1** is narrower (FWHM = 2.6 eV) compared with that of complex **2** (FWHM = 2.9 eV) (Figure S6f). The observed broadening of the corresponding S2p (**1**) and N1s (**2**) BE can be explained by the presence of Hg-S/S-C bonds in complex **1** and Zn-N/N-C bonding in complex **2**. Vice versa, the narrow peaks for N1s in **1** and for S2p in **2** can be referred to the formation of only N-C bonds in Hg(II)-monensinate and S-C in Zn(II)-monensinate, respectively. The results obtained demonstrate that the total difference in binding energy [N1s—S2p] for the target coordination species is 235.5 eV for **1** and 236.0 eV for **2**. This observation corroborates well with some of the available data for metal thiocyanates, where the [N1s—S2p] value is larger for M-NCS than for M-SCN and is used as a discriminator of different SCN-ligand bonding modes [58]. On the other hand, XPS data should be treated with considerable caution as there are cases where the energy difference is the same for related series of complexes containing N- and S-bound thiocyanate [59]. In the present study, despite the questionable reliability of XPS when seeking to usefully study complex coordination systems, we infer that it can be applied as a complementary technique for the structural evaluation of SCN-containing metal complexes.

### 2.4. Antibacterial Properties of MonNa and Complex ***2***

To evaluate the impact of Zn(II) as a biologically relevant metal ion on the activity of sodium monensinate, we conducted an antimicrobial assay using five Gram-positive microorganisms, representative of *Bacillus* (*B. subtilis*, *B. cereus*), *Kocuria* (*K. rhizophila*) and *Staphylococcus* (*S. aureus*, *S. saprophyticus*). Gram-negative bacteria are inherently resistant to polyether ionophorous antibiotics and their complexes, probably due to the size of the molecules with molecular weights above 600 Da [44] and were excluded from the present study.

The effect of Zn(SCN)_2_ on bacterial growth is negligible as the metal salt is not effective at the highest concentration studied (1 mg/mL, 5.6 mM). *K. rhizophila* and *B. cereus* appear to be the most resistant and sensitive strains, respectively, among the microorganisms studied for the effect of MonNa or complex **2** on their growth ability (Table 4). The incorporation of Zn(II) cations into the structure of sodium monensinate preserves or enhances the activity of the parent antibiotic. With the exceptions of *B. cereus* and *S. aureus*, the remaining target bacteria are 2 to 4 times more susceptible to the effect of Zn(II) complex compared with MonNa. In the case of *K. rhizophila,* the two-fold increase in the MIC (µM) of **2** can be explained by the introduction of two moles of antibiotic per one mole of the complex. In contrast, the higher potency of Zn(II) species against the strains of *B. subtilis* and *S. saprophyticus* cannot be attributed to a simple synergic effect of MonNa and Zn(II), while complex **2** retains the inhibitory ability of MonNa towards *B. cereus* and *S. aureus*. In our study, the target bacteria can be arranged in the following hierarchy according to their decreasing susceptibility: *BC* < *SA* ≅ *SS* < *BS* < *KR* (MonNa) and *BC* < *SS* < *SA* < *BS* < *KR* (**2**).

The outcome of the conducted assay reveals that each therapeutic candidate should be investigated as thoroughly as possible, and its effect on a specific bacterial strain cannot be unambiguously transferred to other similar microorganisms. On the other hand, mercury(II) is a toxic ion with no known positive biological functions, although some of its complexes would be potent antimicrobial agents [60,61,62]. At the same time, reducing concentration levels of Hg(II) in animal and human organisms is challenging, so the electroneutral mercury(II)-containing complex **1** can be treated as a possible species formed by coordination with sodium monensinate, especially at heavy metal intoxication in stock farming (animal husbandry).

## 3. Materials and Methods

### 3.1. Materials and Reagents

Sodium monensinate (MonNa, p.a.) was provided by Biovet Ltd. (Peshtera, Bulgaria). The metal(II) thiocyanates (Hg(SCN)_2_, Zn(SCN)_2_)), acetonitrile (MeCN), and methanol (MeOH) (p.a. grade) were purchased from local suppliers, and CDCl_3_ was acquired from Deutero GmbH (Kastellaun, Germany).

### 3.2. Synthesis of ***1*** and ***2***

Metal(II) thiocyanate (0.1 mmol in 2 mL MeOH, 31.7 mg Hg(SCN)_2_ or 18.2 mg Zn(SCN)_2_) was added dropwise to a solution of MonNa (0.1 mmol in 3 mL MeCN:MeOH = 2:1, 69.3 mg). The resulting mixtures were stirred for 30 min. at r. t. and the slow evaporation of the solvent mixtures led to the formation of colourless crystals (**1**) or polycrystalline solids (**2**). These were washed with MeCN, filtered, and dried in an exicator.

MonNa [63], composition C_36_H_61_O_11_Na: ^1^H-NMR (600 MHz, δ (ppm, assignment), CDCl_3_): 4.40 (20CH), 4.02 (5CH), 3.97/3.29 (26CH_2_), 3.93 (17CH), 3.89 (7CH), 3.82 (21CH), 3.53 (13CH), 3.37 (28OCH_3_), 3.18 (3CH), 2.52 (2CH), 2.30/1.46 (15CH_2_), 2.25 (18CH), 2.21 (6CH), 2.18/1.54 (19CH_2_), 2.06 (4CH), 2.00/1.70 (10CH_2),_ 1.97/1.71 (11CH_2_), 1.90/1.68 (8CH_2_), 1.77/1.53 (14CH_2_), 1.59/1.50 (32CH_2_), 1.50 (31CH_3_), 1.45 (24CH), 1.40/1.31 (23CH_2_), 1.35 (22CH), 1.23 (27CH_3_), 1.17 (29CH_3_), 0.94 (33CH_3_), 0.93 (30CH_3_), 0.90 (34CH_3_), 0.84 (36CH_3_), 0.80 (35CH_3_).

Complex **1**, [Hg(MonNa)_2_(SCN)_2_] composition: C_74_H_122_O_22_S_2_N_2_Na_2_Hg, MW 1702.4 g/mol, yield 63.0 mg (74%). Calc. C, 52.21; H, 7.22; N, 1.65; S, 3.77; Na, 2.70; Hg, 11.78%. Found: C, 51.78; H, 6.82; N, 1.82; S, 4.14; Na, 2.50; Hg, 10.96%. ^1^H-NMR (600 MHz, δ (ppm, assignment, multiplicity, *J* [Hz]), CDCl_3_): 4.37 (20CH, m), 4.00 (5CH, dd, *2.1*; *11.1*), 3.95/3.27 (26CH_2_, d, *11.8*), 3.91 (17CH, d, *3.5*), 3.86 (7CH, m), 3.80 (21CH, dd, *3.9; 10.0*), 3.51 (13CH, dd, *4.9; 10.9*), 3.35 (28OCH_3_, s), 3.17 (3CH, dd, *1.8; 11.9*), 2.51 (2CH, m), 2.27/1.44 (15CH_2_, m/m), 2.23 (18CH, m), 2.18 (6CH, m), 2.16/1.51 (19CH_2_, m/m), 2.04 (4CH, m), 1.98/1.67 (10CH_2_, m/m), 1.96/1.69 (11CH_2_, m/m), 1.88/1.67 (8CH_2_, m/m), 1.75/1.53 (14CH_2_, m/m), 1.56/1.48 (32CH_2_, qd, *7.7; 14.4*, qd, *7.0; 14.6*), 1.47 (31CH_3_, s), 1.45 (24CH, m), 1.41/1.30 (23CH_2_, m/m), 1.30 (22CH, m), 1.22 (27CH_3_, d, *6.7*), 1.15 (29CH_3_, d, *6.8*), 0.91 (33CH_3_, t, *7.4*), 0.91 (30CH_3_, d, *7.1*), 0.87 (34CH_3_, d, *6.9*), 0.82 (36CH_3_, d, *6.2*), 0.78 (35CH_3_, d, *6.2*).

Complex **2**, [Zn(MonNa)_2_(NCS)_2_] composition: C_74_H_122_O_22_S_2_N_2_Na_2_Zn, MW 1567.3 g/mol, yield 61.9 mg (79%). Calc. C, 56.71; H, 7.85; N, 1.79; S, 4.09; Na, 2.93; Zn, 4.17%. Found C, 56.95; H, 7.54; N, 1.77; S, 3.85; Na, 2.40; Zn, 4.65%. ^1^H-NMR (600 MHz, δ (ppm, assignment, multiplicity, *J* [Hz]), CDCl_3_): 4.41 (20CH, m), 3.88 (5CH, dd, *2.0; 11.5*), 3.96/3.40 (26CH_2_, d, *12.0*), 3.95 (7CH, m), 3.92 (17CH, d, *3.6*), 3.85 (21CH, dd, *3.7; 10.0*), 3.37 (28OCH_3_, s), 3.52 (3CH, dd, *5.1; 10.9*), 3.27 (13CH, d, *9.6*), 2.58 (2CH, dd, *7.1; 9.3*), 2.28/1.44 (15CH_2_, m/m), 2.26 (18CH, m), 2.17/1.54 (19CH_2_, m/m), 2.09 (6CH, m), 2.06 (4CH, m), 1.98/1.70 (10CH_2_, m/m), 1.92/1.70 (11CH_2_, m/m), 1.92/1.68 (8CH_2_, m/m), 1.77/1.53 (14CH_2_, m/m), 1.61/1.48 (32CH_2_, qd, *7.9; 14.7*, qd, *7.3; 14.6*), 1.51 (31CH_3_, s), 1.51 (24CH, m), 1.51/1.36 (23CH_2_, m/m), 1.40 (22CH, m), 1.25 (27CH_3_, d, *6.6*), 1.11 (29CH_3_, d, *6.7*), 0.94 (33CH_3_, t, *7.6*), 0.93 (30CH_3_, d, *7.1*), 0.90 (36CH_3_, br d), 0.89 (34CH_3_, d, *7.1*), 0.83 (35CH_3_, d, *6.2*).

### 3.3. Methods

#### 3.3.1. X-ray Crystallography

Data collection and structure solution were conducted at the X-ray Crystallographic Facility, Department of Chemistry and Biochemistry, NDSU, Fargo, ND, USA.

A crystal (approximate dimensions 0.17 × 0.16 × 0.07 mm^3^) was mounted on a Bruker APEX-II CCD diffractometer (Billerica, MA, USA) for data collection at 107 K. A preliminary set of cell constants was calculated from the reflections collected from 4 sets of 30 frames. This produced initial orientation matrices determined from 401 reflections. Data collection was carried out using IµS Cu Kα radiation with a frame time of 10 s at higher angles of data collection (detector’s position 2θ = 100.855°) or 5 s for lower angles of data collection (2θ = −34.239°), and with a detector distance of 4.0 cm. A randomly oriented region of reciprocal space was surveyed to the extent of one sphere and a resolution of 0.84 Å. Nineteen ω-scan sections of frames and φ-scan sections with 1.8° width were collected to achieve the desired completeness of 99.8%. Intensity data were corrected for absorption and decay [64]. The final cell constants were calculated from the xyz centroids of 9823 strong reflections from the actual data collection after integration [65].

The structure was solved and refined using the SHELX 2018 [66] set of programs with Olex 2 v.1.5 software package [67]. The structure was solved with SHELXT 2015 [68] program using intrinsic phasing, which provided most of the non-hydrogen atoms, while full-matrix least squares/difference Fourier cycles were performed in order to locate the remaining non-hydrogen atoms. All non-hydrogen atoms were refined with anisotropic displacement parameters. All hydrogen atoms were placed in ideal positions and refined as riding atoms with relative isotropic displacement parameters. The final full matrix least squares refinement converged to R1 = 3.51% and wR2 = 8.53% (F^2^, all data). The data collection parameters and refinement information for the single-crystal X-ray diffraction experiment are summarized in Table 5. Details on the geometrical data can be found in the Supplementary Materials (Tables S3–S6). Images were generated using CrystalMaker Software Ltd., Oxford, England (www.crystalmaker.com, accessed on 14 June 2024).

#### 3.3.2. Physical Measurements

IR spectra were recorded on a Nicolet 6700 FT-IR, Thermo Scientific (Madison, WI, USA) in KBr pellets. ^1^H- (600.18 MHz) and ^13^C- (150.93 MHz) NMR spectra were acquired on a Bruker NEO 600 spectrometer (Ettlingen, Germany). All spectra were recorded in CDCl_3_ at 298.0 ± 0.1K. The residual solvent peaks (^1^H—7.26 ppm and ^13^C—77.16 ppm) were used as internal standards for the ^1^H- and ^13^C-NMR spectra, respectively. The unambiguous assignment of signals was made based on the gradient-enhanced versions of COSY, HSQC, and HMBC experiments. The chemical shift values of the overlapped protons in the complexes have been determined from the HSQC spectra.

The C, H, N, S analysis was performed on an organic elemental analyser vario MACRO cube (Elementar analysensysteme GmbH, Stuttgart, Germany). Sodium content was calculated by AAS on a Perkin Elmer 1100 B (Walthman, MA, USA) using standard stock solution (1000 µg/mL, Merck, Darmstadt, Germany). Working reference solutions were prepared after suitable dilution in appropriate solvent mixture. Mercury(II) and zinc(II) were determined by complexometric titrations at pH 5.5 (acetate buffer) using xylenol orange as an indicator. Hg(II) content was evaluated by back titration with standard solution of zinc acetate, while Zn(II) was quantified by direct titration with standard solution of ethylenediaminetetracetic acid (EDTA).

Electrospray-mass spectrometry (ESI-MS) measurements were performed on a Waters SYNAPT G2-Si ToF high resolution mass spectrometer (HRMS, Milford, MA, USA). The sample was dissolved in methanol and directly injected for analysis in an electro spray ionization positive mode source (ESI+). ESI+ conditions were as follows: capillary potential 3.0 kV, sample cone potential 40 V, temperature source 90 °C, desolvation temperature 250 °C, desolvation gas flow 350 L/h. The observed range was set from 50 to 2000 *m*/*z*. The powder diffraction pattern was obtained on a PANalytical Empyrean X-ray powder diffractometer (Malvern Panalytical, Malvern, UK) with Cu Kα radiation (λ = 1.5418 Å) operating at 40 kV and 30 mA. The X-ray photoelectron spectroscopy (XP, XPS) studies were performed on a VG Escalab II system (Thermo Fisher Scientific, Waltham, MA, USA), using Al Kα radiation with an energy of 1486.6 eV. The chamber pressure was 1.10^−9^ Torr. The C1s line of adventitious carbon at 284.9 eV was used as an internal standard by which to calibrate the binding energies (BE). Photoelectron spectra were corrected by Shirley-type background subtraction and quantified using peak area and Scofield’s photo-ionization cross-section. The accuracy of the measured BE was ±0.2 eV. Spectra were evaluated by CasaXPS software v. 2.3.25PR1.0 and fitted using a mixed Gaussian/Lorentzian product formula with 30% Lorentzian.

#### 3.3.3. Antibacterial Assay

The ability of MonNa and complexes **1**–**2** to inhibit the visible growth of microorganisms was evaluated as their minimum inhibitory concentration (MIC, mg/mL, µM). A series of methanol solutions down to 0.25 µg/mL was prepared by double-dilution method starting from 1 mg/mL. The assay included a set of five Gram-positive non-pathogenic bacteria, supplied by the National Bank for Industrial Microorganisms and Cell Cultures (NBIMCC, Sofia, Bulgaria): *Bacillus subtilis* (*BS*, NBIMCC 1050, ATCC 11774) (ATCC, Manassas, VA, USA), *Bacillus cereus* (*BC*, NBIMCC 1085, ATCC 11778), *Kocuria rhizophila* (*KR*, NBIMCC 159, ATCC 9341), *Staphylococcus aureus* (*SA*, NBIMCC 509, ATCC 6538) and *Staphylococcus saprophyticus* (*SS*, NBIMCC 3348). Nutrient agar (pH 7.2–7.4) containing meat extract (1%), peptone (1%) and NaCl (0.5%) was used as culture media.

The double-layer agar diffusion method was carried out on Petri dishes (90 mm) containing sterile (10 mL) and inoculated (10 mL, 1.5% inoculum, McFarland 4, A_650_ = 0.8–1) agar layers. After media solidification, the holes (punched in 6 mm diameters) were filled with 20 µL of the tested solutions. The diameter of the inhibited zones was read 24 h after incubation at 30 °C. Three separate experiments were performed in triplicate (a total of nine measurements). All equipment and culture media (delivered from local suppliers) were sterile. Methanol served as a negative control.

## 4. Conclusions

The reaction of sodium monensinate (MonNa) with Hg(SCN)_2_ or Zn(SCN)_2_ in acetonitrile–methanol solution leads to the respective formation of new heteronuclear complexes, [Hg(MonNa)_2_(SCN)_2_] and [Zn(MonNa)_2_(NCS)_2_]. The coordination species are isostructural, with the metal(II) ions placed in a distorted tetrahedral environment where two of the positions are occupied by oxygen atoms originating from the parent antibiotic. The other two binding sites involve S-donor atoms in the case of Hg(II) complex and N-donors in its Zn(II) counterpart. The inclusion of biometal ions positively affects the activity of MonNa, while the binding of Hg(II) cations reveals the antidote potential of the antibiotic against heavy metal intoxications that may occur in animal husbandry.

## Figures and Tables

**Table 3:** ^13^C-NMR data of MonNa and complexes **1**–**2** in CDCl_3_.

C Atom	MonNa	Hg(II) Complex, 1		Zn(II) Complex, 2	
	δ, ppm	δ_1_, ppm	Δ_1_, ppm	δ_2_, ppm	Δ_2_, ppm
1C	181.3	181.3	0.0	181.9	−0.6
9C	107.2	107.1	0.1	107.1	0.1
25C	98.4	98.4	0.0	98.6	−0.2
16C	86.0	85.9	0.1	85.9	0.1
12C	85.4	85.3	0.1	85.5	−0.1
17C	85.1	85.1	0.0	84.9	0.2
3C	83.2	83.0	0.2	82.3	0.9
13C	82.7	82.6	0.1	82.2	0.5
20C	76.6	76.5	0.1	76.4	0.2
21C	74.6	74.6	0.0	74.8	−0.2
7C	70.6	70.5	0.1	70.4	0.2
5C	68.5	68.4	0.1	68.3	0.2
26C	65.1	65.0	0.1	65.6	−0.5
28C	58.0	58.0	0.0	58.1	−0.1
2C	45.2	45.0	0.2	44.3	0.9
10C	39.4	39.3	0.1	39.3	0.1
4C	37.6	37.5	0.1	37.5	0.1
24C	36.7	36.6	0.1	36.2	0.5
23C	35.9	35.7	0.2	35.4	0.5
6C	35.0	34.9	0.1	35.2	−0.2
18C	34.5	34.4	0.1	34.3	0.2
8C	33.7	33.6	0.1	33.5	0.2
19C	33.5	33.4	0.1	33.5	0.0
11C	33.4	33.3	0.1	33.3	0.1
22C	32.0	31.9	0.1	31.9	0.1
32C	30.7	30.7	0.0	30.6	0.1
15C	30.0	29.9	0.1	30.2	−0.2
31C	27.6	27.5	0.1	27.6	0.0
14C	27.4	27.4	0.0	27.3	0.1
35C	16.9	16.9	0.0	16.8	0.1
27C	16.9	16.8	0.1	16.4	0.5
36C	16.2	16.2	0.0	16.3	−0.1
34C	14.7	14.7	0.0	14.7	0.0
29C	11.1	11.2	−0.1	11.4	−0.3
30C	10.6	10.6	0.0	10.7	−0.1
33C	8.3	8.3	0.0	8.3	0.0

Δ_1_ = δ_MonNa_ − δ**_1_**; Δ_2_ = δ_MonNa_ − δ**_2_**.

**Table 4:** Effect of tested compounds on bacterial growth.

Compound	Concentration	Bacterial Strain				
		*BS*	*BC*	*KR*	*SA*	*SS*
MonNa	µg/mL	62.50	1.95	125.0	31.25	31.25
	µM	90	2.8	180	45	45
**2**	µg/mL	31.25	3.91	125.0	61.25	15.63
	µM	20	2.5	80	40	10

**Table 5:** Experimental details [65,67,69].

**Crystal Data**	
Chemical formula	C_74_H_122_HgN_2_Na_2_O_22_S_2_
*M*_r_	1702.42
Crystal system, space group	Monoclinic, C_2_
Temperature (K)	107.01
*a*, *b*, *c* (Å)	19.3234(7), 15.4753(5), 13.5120(5)
*V* (Å^3^)	4039.2(2)
*Z*	2
Radiation type, λ [Å]	Cu *K*α, 1.54178
µ (mm^−1^)	4.594
Crystal size (mm^3^)	0.169 × 0.163 × 0.073
**Data Collection**	
Diffractometer	Bruker APEX-II CCD
Absorption correction	Multi-scan
*T*_min_, *T*_max_	0.549, 0.753
No. of measured, independent andobserved [*I* > 2σ(*I*)] reflections	27485, 7140, 7136
*R*_int_	0.0441
Resolution (Å^−1^)	0.84
**Refinement**	
*R*[*F*^2^ > 2σ(*F*^2^)], *wR*(*F*^2^), *S*	0.0351, 0.0883, 1.072
No. of reflections	7140
No. of parameters	477
No. of restraints	1
Δρ_max_, Δρ_min_ (e Å^−3^)	1.86, −0.51
Absolute structure	Flack x determined using quotients [(I+)−(I−)]/[(I+)+(I−)] [70]
Absolute structure parameter	−0.026(5)

## Data Availability

Data are available from the authors upon request.

## Supplementary Materials

The following supporting information can be downloaded at: https://www.mdpi.com/article/10.3390/molecules29133106/s1, Table S1: Orthogonal coordinates [Å] of Zn, O1 and SCN groups in the cell after relaxation of structures **2B** and **2C**; Table S2: Selected bond lengths and angles that undergo major changes during relaxation processes; Table S3: Fractional atomic coordinates (×10^4^) and equivalent isotropic displacement parameters (Å^2^ × 10^3^) for complex **1**. U_eq_ is defined as 1/3 of the trace of the orthogonalized U_IJ_ tensor; Table S4: Bond lengths for complex **1**; Table S5: Bond angles for complex **1**; Table S6: Torsion angles for complex **1**; Figure S1: ^1^H-NMR of complexes **1**–**2** in CDCl_3_; Figure S2: ^13^C-NMR spectra of complexes **1**–**2** in CDCl_3_; Figure S3: ESI-MS+ of Hg(II) complex **1**; Figure S4: ESI-MS+ of Zn(II) complex **2**; Figure S5: Structure of simulated Zn(II) species (a) **2B** and (b) **2C**. H-atoms are omitted for clarity; Figure S6: (a) XPS survey spectra of complexes **1** and **2**; (b−f) high-resolution spectra of C1s, Hg4f, Zn2p, S2p, and N1s. CCDC 2355767 contains the supplementary crystallographic data for this paper. These data can be obtained free of charge via http://www.ccdc.cam.ac.uk (or by an application from the Cambridge Crystallographic Data Centre, 12 Union Road, Cambridge CB2 1EZ, UK; Fax: +44-01223-336033; or e-mail: deposit@ccdc.cam.ac.uk.
