# Supplementary material for: Heteronuclear Complexes of Hg(II) and Zn(II) with Sodium Monensinate as a Ligand
Source: Molecules. 2024 Jun 29;29(13):3106. doi: 10.3390/molecules29133106 (PMC11243498; doi:10.3390/molecules29133106)
Supplement: Supplementary file 1 [file molecules-29-03106-s001.zip › molecules-3040803-supplementary.pdf]

Heteronuclear Complexes of Hg(II) and Zn(II) with Sodium Monensinate as a Ligand

Ivayla Pantcheva [1*], Nikolay Petkov [1*], Elzhana Encheva [1,2], Stiliyan Kolev [1], Svetlana Simova [3], Aleksandar Tsanev [4], Petar Dorkov [5] and Angel Ugrinov [6]

[1] Faculty of Chemistry and Pharmacy, Sofia University "St. Kliment Ohridski", 1164 Sofia, Bulgaria; eencheva@ipc.bas.bg (E.E.), stiliiankolev@gmail.com (S.K.)
[2] Institute of Physical Chemistry, Bulgarian Academy of Sciences, 1113 Sofia, Bulgaria; eencheva@ipc.bas.bg
[3] Institute of Organic Chemistry with Centre of Phytochemistry, Bulgarian Academy of Sciences, 1113 Sofia, Bulgaria; Svetlana.Simova@orgchm.bas.bg
[4] Institute of General and Inorganic Chemistry, Bulgarian Academy of Sciences, 1113 Sofia, Bulgaria; tsanew@abv.bg
[5] Research and Development Department, Biovet Ltd., 4550 Peshtera, Bulgaria; p_dorkov@biovet.com
[6] Department of Chemistry and Biochemistry, North Dakota State University, Fargo ND, USA; angel.ugrinov@ndsu.edu
* Correspondence: ahnp@chem.uni-sofia.bg (N.P.); ipancheva@chem.uni-sofia.bg (I.P.); Tel.: +359-2-8161446 (N.P., I.P.)

Supporting information

Table S1. Orthogonal coordinates [Å] of Zn, O1 and SCN-group in the cell after relaxation of structures **2B** and **2C**.

Atom	Relaxation of 2B (step 3)			Relaxation of 2C (step 4)		
	x	y	z	x	y	z
Zn01	−6.661	19.685	-2.298	5.375	19.256	5.890
O1	−4.970	18.338	-1.864	4.105	18.250	4.699
O1	−8.295	18.294	-2.807	6.619	18.070	6.933
S1	−6.192	20.155	-4.604	3.718	22.189	7.116
S1	−7.146	20.009	0.030	7.104	22.286	5.062
C	−5.704	21.910	-4.191	3.542	20.591	7.888
C	−7.708	21.762	-0.288	7.242	20.801	4.084
N1	−5.019	22.796	-4.566	6.033	20.062	4.365
N1	−8.427	22.598	0.133	3.782	15.119	4.618

Table S2. Selected bond lengths and angle, that undergo major changes during relaxation processes.

Bond lengths	SCXRD of 1	Relaxation of 2B	Relaxation of 2C
Hg-O1	2.43 Å	–	–
Hg-S1	2.38 Å	–	–
Zn01-O1	–	2.21 Å	2.01 Å
Zn01-S1	–	2.40 Å	2.40 Å
Zn01-N1	–	–	1.85 Å
**Bond angle**			
S1-C-N1	175.9°	141.8°	104.7°

Table S3. Fractional atomic coordinates (× 10^4) and equivalent isotropic displacement parameters ($Å^2 \times 10^3$) for complex **1**. U_{eq} is defined as 1/3 of the trace of the orthogonalized U_{IJ} tensor.

Atom	*x*	*y*	*z*	U(eq)
Hg01	5000	7527.5(2)	5000	21.46(11)
S1	6176.1(10)	7805.4(15)	4644.5(14)	45.2(5)
Na1	4213.8(12)	4006.7(17)	2570.5(19)	22.0(5)
O7	3357(2)	3211(3)	3511(3)	23.6(9)
O8	4287.0(17)	2502(5)	2153(2)	22.4(7)
O2	5163(2)	6327(3)	2033(3)	26.6(9)
O3	3980(3)	8650(3)	1542(3)	27.2(9)
O9	5393(2)	3414(3)	2684(3)	19.4(9)
O5	4257(2)	4622(3)	1001(3)	24.6(9)
O1	4884(2)	6546(3)	3603(3)	25.3(9)

Table S3. Fractional atomic coordinates (× 10^4) and equivalent isotropic displacement parameters (Å^2 × 10^3) for complex **1**. U_{eq} is defined as 1/3 of the trace of the orthogonalized U_{IJ} tensor.

**Atom**	***x***	***y***	***z***	**U(eq)**
O6	3117(2)	4635(3)	2314(3)	21.0(8)
O10	5628(2)	4731(3)	1957(3)	22.4(8)
C19	5154(4)	1463(4)	2568(6)	26.0(15)
O4	2835(2)	5967(3)	1588(3)	21.0(8)
C4	3489(3)	7236(4)	1783(5)	22.6(12)
C16	3398(3)	2272(4)	3357(5)	22.7(14)
C6	3814(5)	6053(5)	495(6)	23.6(16)
C2	4723(3)	7717(3)	2438(4)	20.6(13)
C29	3218(3)	7368(4)	2819(4)	23.8(15)
C21	5459(3)	2939(4)	1778(5)	22.7(12)
C15	2861(4)	2083(5)	2513(5)	30.8(14)
C30	3433(3)	6519(4)	-352(5)	27.9(13)
C26	5598(3)	4580(4)	3745(5)	23.4(12)
C3	4158(3)	7757(3)	1602(4)	21.4(13)
C8	3058(3)	4732(4)	540(4)	22.7(11)
C1	4939(3)	6793(4)	2703(5)	21.6(12)
C14	2448(4)	2916(5)	2383(5)	31.4(14)
C11	1945(3)	4792(5)	2772(5)	27.0(13)
C34	4045(4)	604(5)	2404(6)	36.1(16)
C5	3535(3)	6271(4)	1525(4)	20.6(11)
C27	5365(3)	8213(5)	2105(5)	28.9(13)
C25	5803(3)	4186(4)	2756(4)	20.6(11)
C17	4157(3)	2127(4)	3116(5)	24.2(12)
C18	4465(4)	1230(4)	3049(6)	28.8(14)
C23	6679(3)	3449(4)	1750(5)	25.6(12)
C9	2763(3)	5056(4)	1506(4)	20.8(11)
C31	2939(3)	4918(4)	4046(5)	26.7(13)
C22	6212(3)	2655(5)	1620(5)	24.6(15)
C13	2661(3)	3473(4)	3277(5)	24.7(12)
C24	6572(3)	3936(4)	2710(5)	22.8(12)
C36	7059(3)	4725(5)	2787(6)	32.3(14)
C12	2659(3)	4457(4)	3121(4)	22.1(11)
C28	3748(5)	8919(6)	593(7)	33(2)
C20	4955(3)	2191(4)	1836(5)	23.6(12)
O11	4882(2)	4804(3)	3713(3)	24.5(9)
C33	3647(4)	2028(5)	5209(6)	41.1(17)
C32	3205(4)	1804(4)	4313(5)	31.8(14)
C35	6297(4)	2236(5)	612(5)	33.8(15)
C10	2002(3)	4860(4)	1654(5)	24.4(12)
N1	6336(6)	9485(7)	5409(9)	89(4)
C7	3785(3)	5079(4)	346(4)	22.1(11)
C37	6247(5)	8794(8)	5107(7)	62(3)

Table S4. Bond lengths for complex **1.**

Atom	Atom	Length/Å	Atom	Atom	Length/Å
Hg01	S1[1]	2.3746(18)	C4	C5	1.536(8)
Hg01	S1	2.3746(18)	C16	C15	1.550(9)
Hg01	O1	2.429(4)	C16	C17	1.526(9)
Hg01	O1[1]	2.429(4)	C16	C32	1.535(10)
S1	C37	1.657(13)	C6	C30	1.525(11)
Na1	O7	2.447(5)	C6	C5	1.543(10)
Na1	O8	2.400(8)	C6	C7	1.522(10)
Na1	O9	2.457(5)	C2	C3	1.551(8)
Na1	O5	2.328(5)	C2	C1	1.529(8)
Na1	O6	2.350(5)	C2	C27	1.536(8)
Na1	C25	3.086(6)	C21	C22	1.539(8)
Na1	O11	2.339(5)	C21	C20	1.517(9)
O7	C16	1.471(9)	C15	C14	1.524(10)
O7	C13	1.432(7)	C26	C25	1.531(8)
O8	C17	1.453(7)	C26	O11	1.425(7)
O8	C20	1.452(7)	C8	C9	1.525(8)
O2	C1	1.244(8)	C8	C7	1.532(8)
O3	C3	1.427(7)	C14	C13	1.530(9)
O3	C28	1.409(10)	C11	C12	1.537(8)
O9	C21	1.437(8)	C11	C10	1.521(9)
O9	C25	1.435(7)	C34	C18	1.523(11)
O5	C7	1.441(7)	C25	C24	1.539(8)
O1	C1	1.281(8)	C17	C18	1.514(9)
O6	C9	1.429(7)	C23	C22	1.532(9)
O6	C12	1.448(7)	C23	C24	1.519(9)
O10	C25	1.405(7)	C9	C10	1.520(8)
C19	C18	1.540(10)	C31	C12	1.526(8)
C19	C20	1.542(10)	C22	C35	1.522(9)
O4	C5	1.436(7)	C13	C12	1.538(8)
O4	C9	1.420(7)	C24	C36	1.543(9)
C4	C29	1.521(8)	C33	C32	1.504(11)
C4	C3	1.550(8)	N1	C37	1.157(15)

[1]1-X,+Y,1-Z

Table S5. Bond angles for complex **1.**

Atom	Atom	Atom	Angle/˚	Atom	Atom	Atom	Angle/˚
S1[1]	Hg01	S1	159.13(11)	C14	C15	C16	105.3(5)
S1[1]	Hg01	O1[1]	91.42(12)	O11	C26	C25	110.1(5)
S1	Hg01	O1[1]	101.64(11)	O3	C3	C4	108.1(5)
S1[1]	Hg01	O1	101.64(11)	O3	C3	C2	104.1(4)
S1	Hg01	O1	91.42(12)	C4	C3	C2	116.0(5)
O1	Hg01	O1[1]	102.6(2)	C9	C8	C7	113.3(5)
C37	S1	Hg01	99.2(3)	O2	C1	O1	123.8(6)
O7	Na1	O9	114.75(19)	O2	C1	C2	118.0(5)
O7	Na1	C25	133.52(19)	O1	C1	C2	118.2(5)
O8	Na1	O7	71.28(15)	C15	C14	C13	104.9(5)

Table S5. Bond angles for complex **1.**

Atom	Atom	Atom	Angle/°	Atom	Atom	Atom	Angle/°
O8	Na1	O9	65.93(15)	C10	C11	C12	103.9(5)
O8	Na1	C25	92.37(16)	O4	C5	C4	104.2(5)
O9	Na1	C25	27.09(15)	O4	C5	C6	109.5(5)
O5	Na1	O7	136.08(18)	C4	C5	C6	116.2(6)
O5	Na1	O8	100.28(17)	O9	C25	Na1	51.2(3)
O5	Na1	O9	98.85(17)	O9	C25	C26	103.6(5)
O5	Na1	O6	75.56(17)	O9	C25	C24	108.7(5)
O5	Na1	C25	88.70(16)	O10	C25	Na1	76.9(3)
O5	Na1	O11	110.67(18)	O10	C25	O9	109.1(5)
O6	Na1	O7	70.39(16)	O10	C25	C26	111.6(5)
O6	Na1	O8	115.15(16)	O10	C25	C24	109.4(5)
O6	Na1	O9	174.4(2)	C26	C25	Na1	80.0(3)
O6	Na1	C25	150.07(18)	C26	C25	C24	114.1(5)
O11	Na1	O7	106.99(18)	C24	C25	Na1	159.1(4)
O11	Na1	O8	129.19(18)	O8	C17	C16	108.8(5)
O11	Na1	O9	70.16(17)	O8	C17	C18	103.6(6)
O11	Na1	O6	111.09(18)	C18	C17	C16	122.0(5)
O11	Na1	C25	50.81(16)	C34	C18	C19	111.2(6)
C16	O7	Na1	112.5(4)	C17	C18	C19	99.1(5)
C13	O7	Na1	112.8(4)	C17	C18	C34	114.4(6)
C13	O7	C16	107.6(5)	C24	C23	C22	113.8(5)
C17	O8	Na1	99.5(4)	O6	C9	C8	108.7(5)
C20	O8	Na1	116.7(4)	O6	C9	C10	104.7(5)
C20	O8	C17	107.9(5)	O4	C9	O6	110.5(4)
C28	O3	C3	114.1(5)	O4	C9	C8	110.8(5)
C21	O9	Na1	104.0(3)	O4	C9	C10	106.4(5)
C25	O9	Na1	101.7(3)	C10	C9	C8	115.6(5)
C25	O9	C21	114.9(5)	C23	C22	C21	108.1(5)
C7	O5	Na1	136.3(4)	C35	C22	C21	111.7(5)
C1	O1	Hg01	122.9(4)	C35	C22	C23	111.5(5)
C9	O6	Na1	135.5(3)	O7	C13	C14	104.2(5)
C9	O6	C12	111.9(4)	O7	C13	C12	108.0(5)
C12	O6	Na1	112.1(3)	C14	C13	C12	116.7(5)
C18	C19	C20	103.7(5)	C25	C24	C36	112.8(5)
C9	O4	C5	114.3(4)	C23	C24	C25	108.2(5)
C29	C4	C3	112.6(5)	C23	C24	C36	110.8(5)
C29	C4	C5	111.3(5)	O6	C12	C11	105.4(5)
C5	C4	C3	114.6(5)	O6	C12	C31	108.6(5)
O7	C16	C15	104.6(5)	O6	C12	C13	107.0(5)
O7	C16	C17	103.4(5)	C11	C12	C13	112.0(5)
O7	C16	C32	109.3(5)	C31	C12	C11	112.9(5)
C17	C16	C15	116.3(6)	C31	C12	C13	110.6(5)
C17	C16	C32	111.5(5)	O8	C20	C19	105.2(5)
C32	C16	C15	111.0(6)	O8	C20	C21	109.9(6)
C30	C6	C5	113.6(6)	C21	C20	C19	116.1(5)

Table S5. Bond angles for complex **1.**

Atom	Atom	Atom	Angle/°	Atom	Atom	Atom	Angle/°
C7	C6	C30	110.7(6)	C26	O11	Na1	114.2(3)
C7	C6	C5	108.9(6)	C33	C32	C16	115.1(6)
C1	C2	C3	112.9(4)	C9	C10	C11	103.8(5)
C1	C2	C27	108.6(5)	O5	C7	C6	112.6(5)
C27	C2	C3	109.0(5)	O5	C7	C8	106.9(5)
O9	C21	C22	111.7(5)	C6	C7	C8	110.9(5)
O9	C21	C20	105.9(5)	N1	C37	S1	175.9(10)
C20	C21	C22	113.6(5)				

[1]1-X,+Y,1-Z

Table S6. Torsion angles for complex **1.**

A	B	C	D	Angle/°	A	B	C	D	Angle/°
Hg01	O1	C1	O2	149.2(5)	C3	C2	C1	O2	57.4(7)
Hg01	O1	C1	C2	−30.1(6)	C3	C2	C1	O1	−123.3(6)
Na1	O7	C16	C15	−94.2(5)	C8	C9	C10	C11	150.7(5)
Na1	O7	C16	C17	28.1(6)	C1	C2	C3	O3	174.0(5)
Na1	O7	C16	C32	147.0(4)	C1	C2	C3	C4	55.3(7)
Na1	O7	C13	C14	86.7(5)	C14	C13	C12	O6	−57.2(7)
Na1	O7	C13	C12	−38.0(5)	C14	C13	C12	C11	57.8(7)
Na1	O8	C17	C16	70.7(5)	C14	C13	C12	C31	−175.2(5)
Na1	O8	C17	C18	−158.2(4)	C5	O4	C9	O6	63.5(6)
Na1	O8	C20	C19	121.9(4)	C5	O4	C9	C8	−57.0(6)
Na1	O8	C20	C21	−3.8(6)	C5	O4	C9	C10	176.6(5)
Na1	O9	C21	C22	169.5(4)	C5	C4	C3	O3	160.5(5)
Na1	O9	C21	C20	−66.4(5)	C5	C4	C3	C2	−83.0(6)
Na1	O9	C25	O10	−54.1(5)	C5	C6	C7	O5	−67.1(7)
Na1	O9	C25	C26	65.0(4)	C5	C6	C7	C8	52.7(7)
Na1	O9	C25	C24	−173.4(4)	C27	C2	C3	O3	−65.2(6)
Na1	O5	C7	C6	90.7(7)	C27	C2	C3	C4	176.1(5)
Na1	O5	C7	C8	−31.3(7)	C27	C2	C1	O2	−63.5(7)
Na1	O6	C9	O4	−95.5(5)	C27	C2	C1	O1	115.8(6)
Na1	O6	C9	C8	26.3(7)	C25	O9	C21	C22	59.3(7)
Na1	O6	C9	C10	150.3(4)	C25	O9	C21	C20	−176.6(5)
Na1	O6	C12	C11	−172.0(4)	C25	C26	O11	Na1	24.9(5)
Na1	O6	C12	C31	66.8(5)	C17	O8	C20	C19	11.0(7)
Na1	O6	C12	C13	−52.6(5)	C17	O8	C20	C21	−114.7(6)
Na1	C25	C24	C23	42.8(13)	C17	C16	C15	C14	−124.2(6)
Na1	C25	C24	C36	165.7(9)	C17	C16	C32	C33	55.4(8)
O7	C16	C15	C14	−10.8(7)	C18	C19	C20	O8	17.5(7)
O7	C16	C17	O8	−68.9(7)	C18	C19	C20	C21	139.2(6)
O7	C16	C17	C18	170.8(6)	C9	O6	C12	C11	0.9(6)
O7	C16	C32	C33	−58.3(8)	C9	O6	C12	C31	−120.4(5)
O7	C13	C12	O6	59.7(6)	C9	O6	C12	C13	120.2(5)
O7	C13	C12	C11	174.8(5)	C9	O4	C5	C4	−172.0(5)
O7	C13	C12	C31	−58.3(6)	C9	O4	C5	C6	63.1(7)
O8	C17	C18	C19	44.9(6)	C9	C8	C7	O5	74.6(6)

Table S6. Torsion angles for complex **1.**

A	B	C	D	Angle/°	A	B	C	D	Angle/°
O8	C17	C18	C34	−73.4(7)	C9	C8	C7	C6	−48.6(7)
O9	C21	C22	C23	−50.9(7)	C22	C21	C20	O8	170.2(5)
O9	C21	C22	C35	−174.0(6)	C22	C21	C20	C19	51.0(7)
O9	C21	C20	O8	47.2(6)	C22	C23	C24	C25	−55.9(6)
O9	C21	C20	C19	−72.0(7)	C22	C23	C24	C36	−180.0(5)
O9	C25	C24	C23	57.4(6)	C13	O7	C16	C15	30.7(7)
O9	C25	C24	C36	−179.7(5)	C13	O7	C16	C17	153.0(5)
O6	C9	C10	C11	31.1(6)	C13	O7	C16	C32	−88.2(6)
O10	C25	C24	C23	−61.6(6)	C24	C23	C22	C21	52.0(7)
O10	C25	C24	C36	61.3(7)	C24	C23	C22	C35	175.2(5)
O4	C9	C10	C11	−85.9(6)	C12	O6	C9	O4	94.0(5)
C16	O7	C13	C14	−38.0(6)	C12	O6	C9	C8	−144.2(5)
C16	O7	C13	C12	−162.8(5)	C12	O6	C9	C10	−20.2(6)
C16	C15	C14	C13	−11.0(7)	C12	C11	C10	C9	−30.3(6)
C16	C17	C18	C19	167.7(6)	C28	O3	C3	C4	-84.9(7)
C16	C17	C18	C34	49.3(9)	C28	O3	C3	C2	151.1(6)
C29	C4	C3	O3	−71.0(6)	C20	O8	C17	C16	−167.1(5)
C29	C4	C3	C2	45.5(7)	C20	O8	C17	C18	−36.0(7)
C29	C4	C5	O4	60.4(6)	C20	C19	C18	C34	83.1(6)
C29	C4	C5	C6	−179.1(6)	C20	C19	C18	C17	−37.5(7)
C21	O9	C25	Na1	111.6(5)	C20	C21	C22	C23	−170.6(5)
C21	O9	C25	O10	57.5(6)	C20	C21	C22	C35	66.4(7)
C21	O9	C25	C26	176.5(5)	O11	C26	C25	Na1	−17.2(4)
C21	O9	C25	C24	−61.8(6)	O11	C26	C25	O9	−63.0(6)
C15	C16	C17	O8	45.1(8)	O11	C26	C25	O10	54.3(6)
C15	C16	C17	C18	−75.2(8)	O11	C26	C25	C24	178.9(5)
C15	C16	C32	C33	−173.2(6)	C32	C16	C15	C14	106.9(6)
C15	C14	C13	O7	29.7(7)	C32	C16	C17	O8	173.7(5)
C15	C14	C13	C12	148.7(6)	C32	C16	C17	C18	53.4(8)
C30	C6	C5	O4	64.6(7)	C10	C11	C12	O6	18.7(6)
C30	C6	C5	C4	−53.0(8)	C10	C11	C12	C31	137.1(5)
C30	C6	C7	O5	167.4(5)	C10	C11	C12	C13	−97.3(6)
C30	C6	C7	C8	−72.9(7)	C7	C6	C5	O4	−59.2(7)
C26	C25	C24	C23	172.5(5)	C7	C6	C5	C4	−176.8(6)
C26	C25	C24	C36	−64.6(7)	C7	C8	C9	O6	−72.7(6)
C3	C4	C5	O4	−170.4(5)	C7	C8	C9	O4	48.9(7)
C3	C4	C5	C6	−49.9(7)	C7	C8	C9	C10	169.9(5)

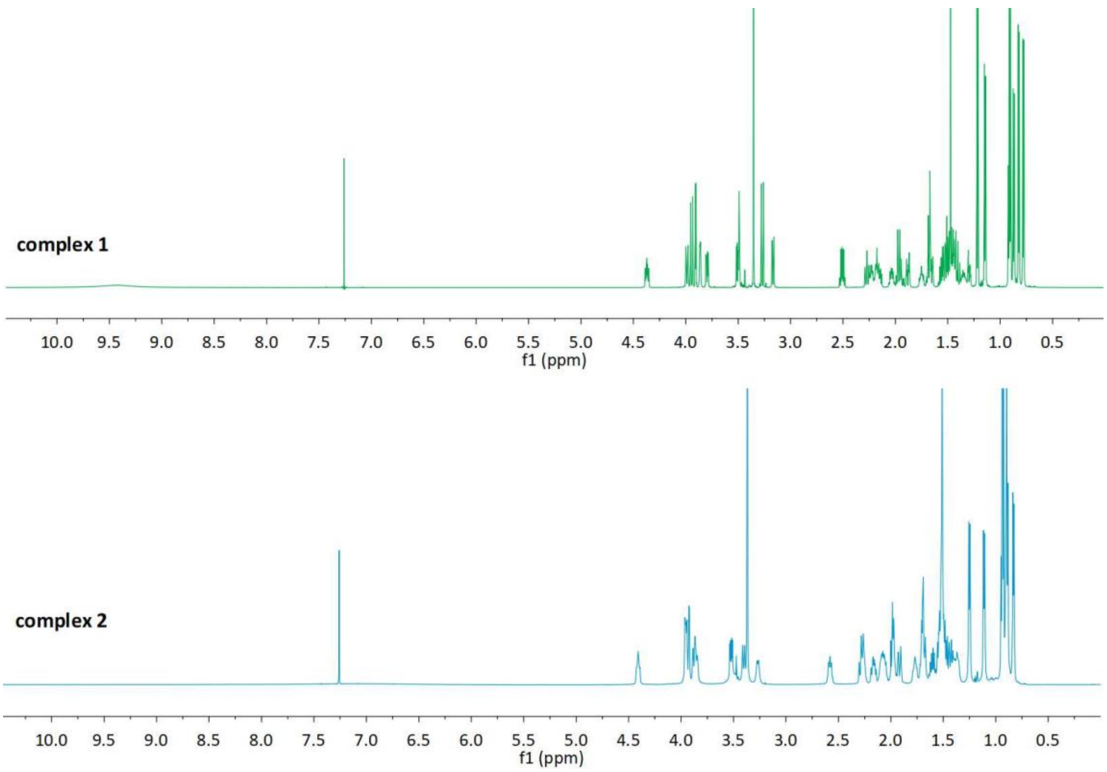

Figure S1. ^{1}H-NMR spectra of complexes **1** and **2** in $CDCl_3$.

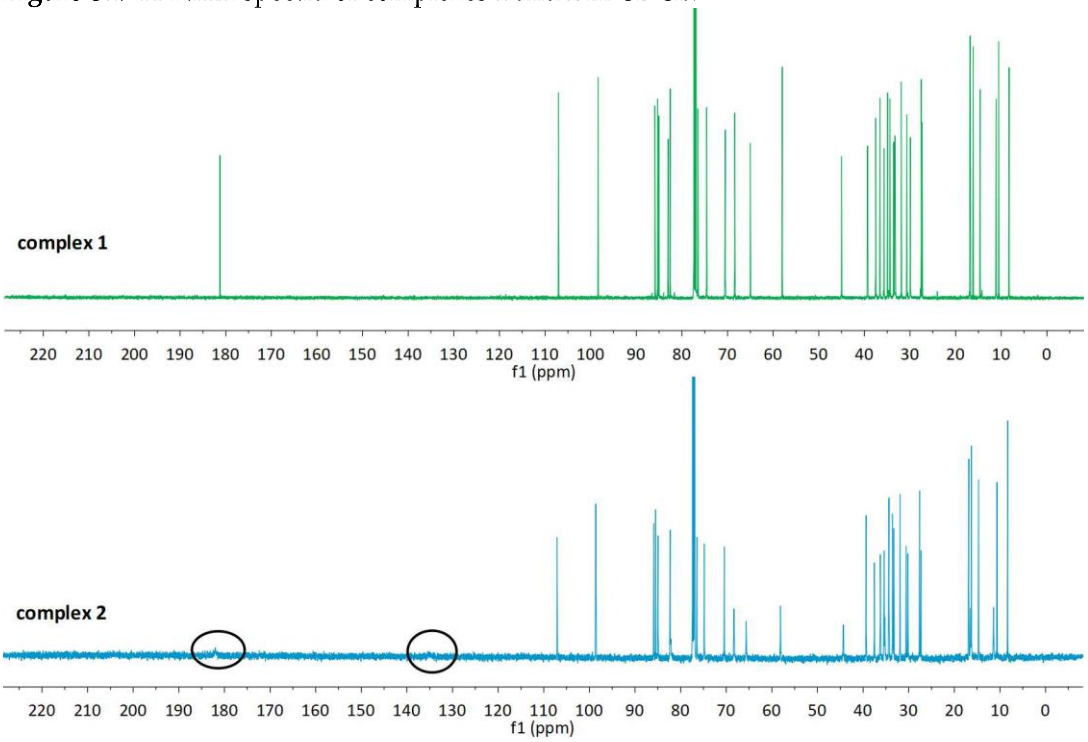

Figure S2. ^{13}C-NMR spectra of complexes **1** and **2** in $CDCl_3$. The signals of the quaternary carbons with a circle are an indication of a long relaxation time and/or exchange.

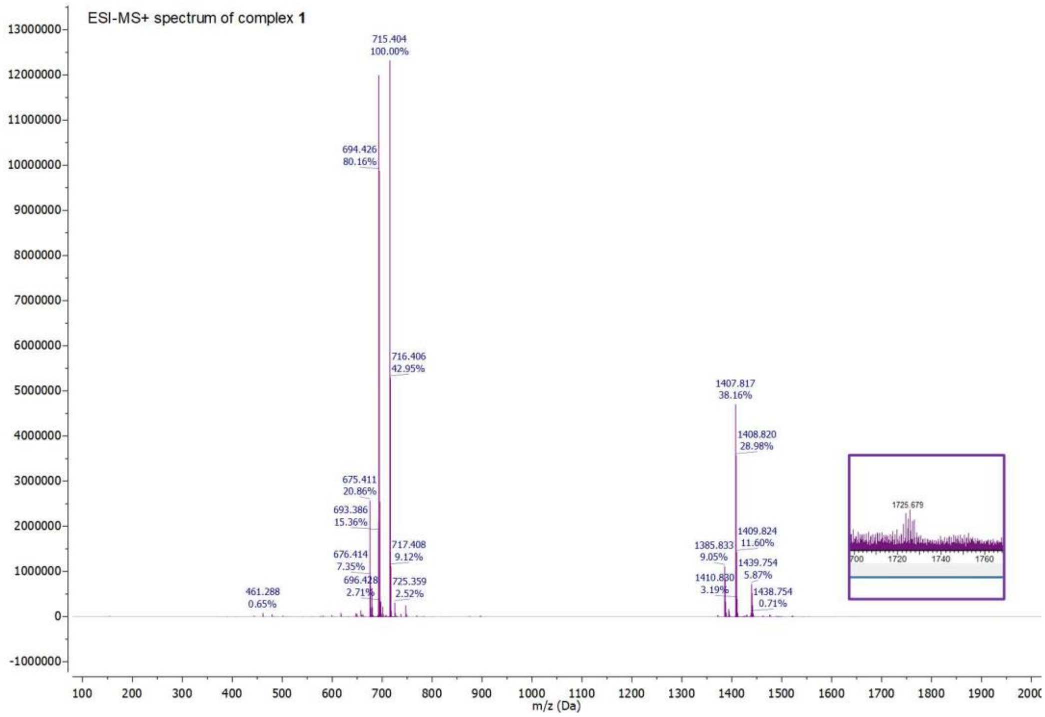

Figure S3. ESI-MS+ of complex **1**. The inset represents the molecular ion $[Hg(MonNa)_2(SCN)_2]Na^+$, observable at significant spectrum magnification.

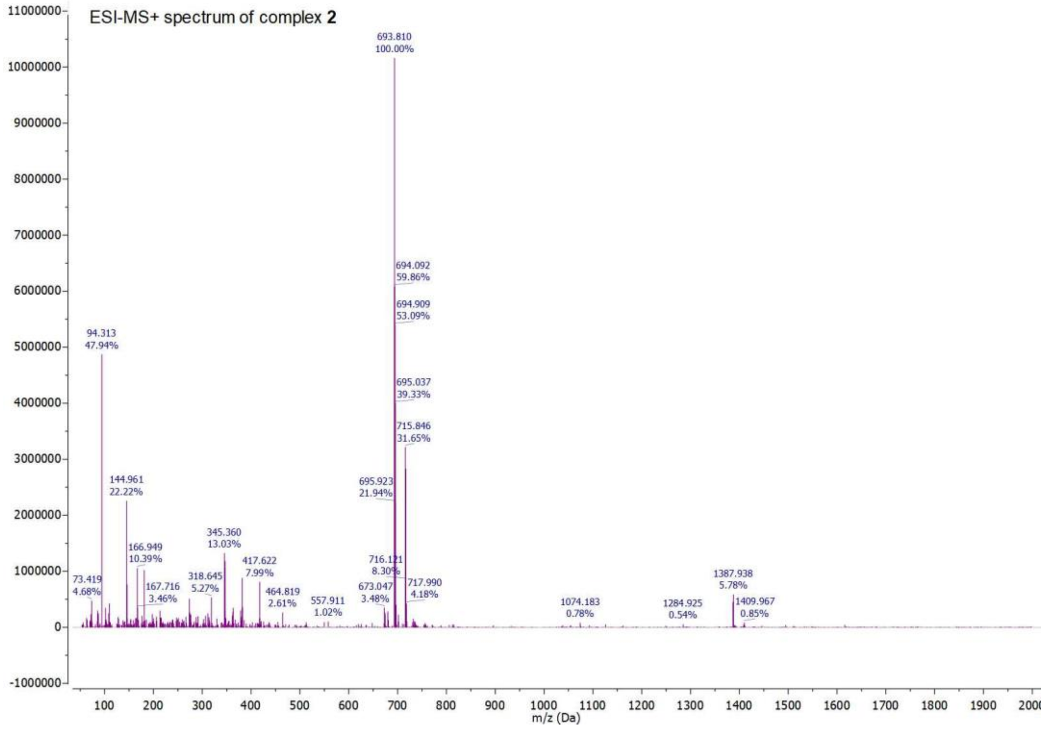

Figure S4. ESI-MS+ of Zn(II) complex **2**.

Details for powder diffraction patterns and Zn(II)-crystal structure simulations

CrystalMaker v.11.0.2 is used to simulate the Zn(II) structure and all powder patterns, presented in Figure 5 of the manuscript. The protocol includes the following:

Step 1. SCXRD structure of Hg(II) complex **1** is used to simulate its powder pattern (**1**, green).

Step 2. Hg-atom was substituted with Zn-atom (**2A**) and powder pattern simulation was made (**2A**, yellow). Only the type of the atom is changed, no manual changes are made to coordinates.

Step 3. Structure **2A** was relaxed with provided from CrystalMaker v.11.0.2 algorithm to obtain **2B** (Figure S5a, Tables S1 and S2). CrystalMaker uses a "Force-Field" calculation employing a hybrid Monte Carlo and least-squares technique with high-quality parameterized potentials to minimize energy and optimize structure (details are available in Chapter 12 of CrystalMaker's manual). Powder pattern was simulated after relaxation (**2B**, red).

Step 4. Structure **2B** was used to exchange the sulphur and nitrogen atoms of the SCN-groups (**2C**, Figure S5b, Tables S1 and S2). After a new relaxation with CryslaMaker software, the powder pattern was simulated (**2C**, purple).

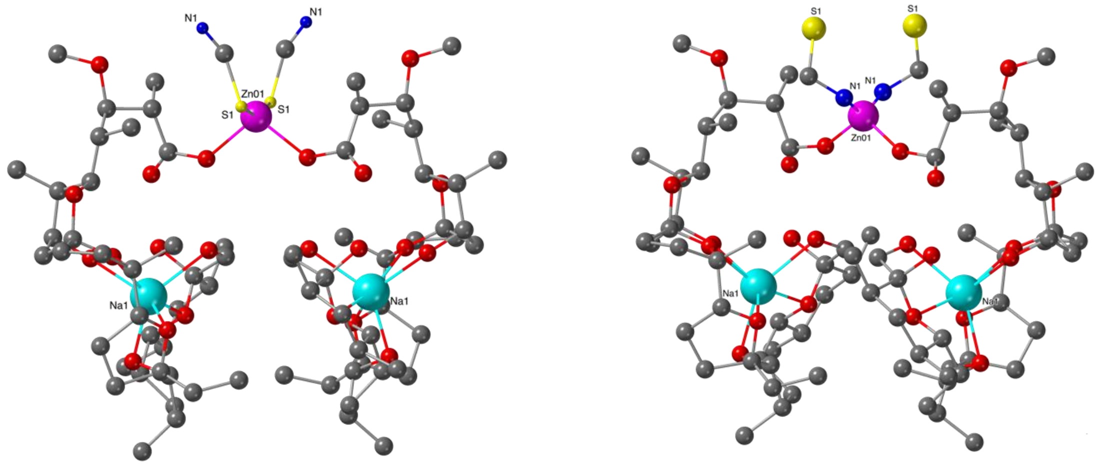

(a) (b)

Figure S5. Structure of simulated Zn(II) species (a) **2B**, (b) **2C**. H-atoms are omitted for clarity.

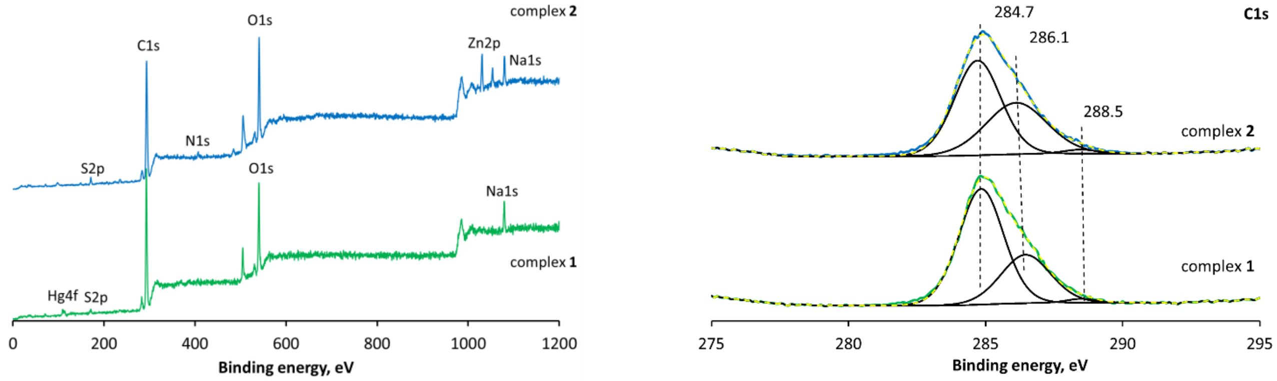

(a) (b)

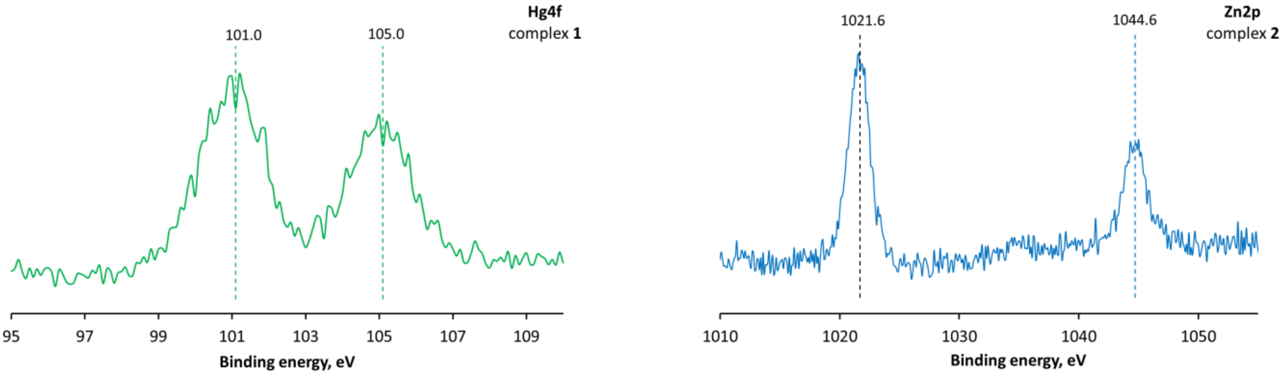

(c) (d)

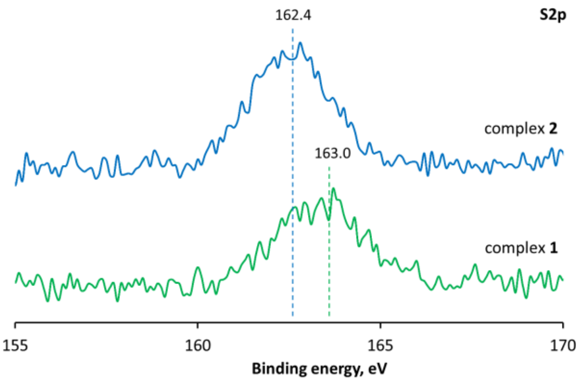

(e)

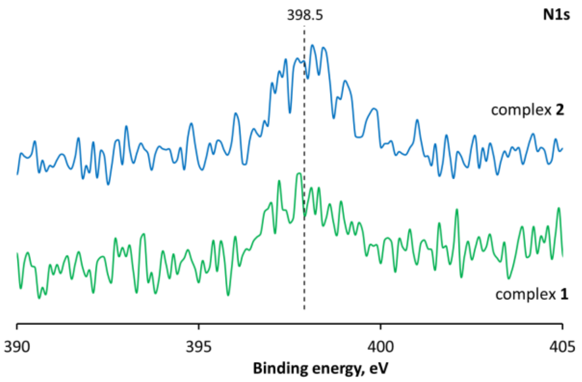

(f)

Figure S6. (a) XPS survey spectra of complexes **1** and **2**; (b–f) High-resolution spectra of C1s, Hg4f, Zn2p, S2p, and N1s.
